# Editorial: Effects of indoor plants on well-being

**DOI:** 10.3389/fpsyg.2024.1483441

**Published:** 2024-09-19

**Authors:** Ke-Tsung Han

**Affiliations:** Department of Landscape Architecture, National Chin-Yi University of Technology, Taichung, Taiwan

**Keywords:** wellbeing, complaint, privacy, attractiveness, satisfaction, physiological adjustment, fractal pattern

The United Nations estimates that 55% of the world's population currently lives in cities, and this urban population is projected to increase by 68% by 2050. As urban residents spend ~80% of their lives indoors, they often lack access to outdoor nature, which is known to be beneficial for health and wellbeing. Although plants are widely recognized as a key representation of nature, research specifically focusing on the impact of indoor plants is relatively limited compared to the extensive studies on the broader effects of nature on health. Therefore, the potential health and wellbeing benefits of indoor plants deserve more investigation. Not only is nature essential in urban environments, but incorporating plants into indoor spaces is also crucial for enhancing people's health and wellbeing.

Recently, two systematic reviews and one systematic review with meta-analyses have examined the influence of indoor plants on health and wellbeing. Han and Ruan (2020), after reviewing 95 Chinese and English journal articles, found that indoor plants primarily affected air characteristics by reducing pollutant concentrations, particularly formaldehyde, benzene, and toluene. They also found that indoor plants increased relative humidity and reduced air temperature. Han and Ruan (2019), based on a review of 50 Chinese and English journal articles, concluded that indoor plants most significantly impacted occupants' self-reported perceptions by increasing positive emotions and reducing negative feelings, as well as reducing physical discomfort. Han et al. (2022), after reviewing 42 Chinese and English journal articles, determined that indoor plants positively affected occupants' objective functions, notably by promoting relaxed physiology and enhanced cognition. Additionally, meta-analyses of 16 studies revealed that indoor plants significantly benefited participants' diastolic blood pressure (SMD = −2.526, 95% CI −4.142, −0.909) and academic achievement (SMD = 0.534, 95% CI 0.167, 0.901).

This Research Topic aimed to deepen the understanding of interactions between indoor plants and humans in various contexts. The Research Topic included four articles, ranging from broader perspectives to specific viewpoints. Dreyer et al. investigated the impact of a certified “green” office building on the wellbeing of 213 employees in Canada. Their findings revealed that: (1) physical features (e.g., air quality and light) enhanced hedonic wellbeing, while social features (e.g., privacy) helped to prevent negative wellbeing; (2) both physical and social features equally predicted eudaimonic wellbeing; (3) a view of the outdoors was positively related to wellbeing, although this effect diminished when other environmental features were taken into account; and (4) person-environment fit, pro-environmental behavior, and social belonging were crucial mediators in the relationship between indoor environmental features and both hedonic and eudaimonic wellbeing.

de Vries et al. conducted a before-and-after control impact quasi-experiment to investigate the effects of plants in the workspace on the health, wellbeing, and functioning of office workers. Five hundred and ninety-four participants from nine organizations in the Netherlands completed the pre-measurement 1 month before the introduction of the plants. Out of these, 254 participants completed both the pre-measurement and the first-round post-measurement ~3 months after the plant introduction. Additionally, 128 participants completed both the pre-measurement and the second-round post-measurement, which was conducted at least 4 months after the plant introduction. The results indicated that, following the introduction of plants into the workspace: (1) complaints about dry air decreased, and both the sense of privacy and the attractiveness of the workspace improved during the first-round post-measurement; and (2) health-related complaints, particularly those experienced at work, decreased, while satisfaction with the workspace increased during the second-round post-measurement.

Ikei et al. conducted a crossover experiment to examine the physiological effects of viewing fresh roses in a vase for 4 min on sympathetic nervous activity in 214 Japanese participants. They found that the initial value of the natural logarithm of the low-frequency to high-frequency ratio [ln(LF/HF)] of heart rate variability (HRV) during the control viewing (without fresh roses) was significantly and negatively correlated with the change in ln(LF/HF) of HRV during visual stimulation with fresh roses. This indicated that participants with high initial sympathetic nervous activity showed a decrease in activity after viewing the roses, whereas those with low initial sympathetic nervous activity exhibited an increase. That is, short-term visual stimulation with fresh roses facilitating physiological adjustment.

Robles et al. aimed to incorporate the benefits of natural design into man-made spaces and conducted two experiments to explore the optimal installation of fractals—self-similar patterns found throughout nature—to maximize their effectiveness in eliciting various psychological experiences within a space. The first experiment involved 94 college students in the USA, who viewed 18 randomized blocks of 20 images and rated their complexity, appeal, naturalness, interest, relaxation, and excitement using a sliding scale. Each block featured one pattern type: fractal, average non-fractal, or large non-fractal. The second experiment included 40 participants from the USA or the UK, who viewed 12 randomized blocks of 40 images and rated the same six factors using a sliding scale. In this experiment, half of the blocks contained fractal images framed with a Euclidean frame, while the other half presented images without the frame. Robles et al. concluded that by controlling the perceived pattern complexity and whether emphasis was placed on pattern boundaries, fractal patterns could predictably influence psychological experiences in man-made spaces, thereby introducing the benefits of nature into built environments.

This Research Topic is just the starting point to explore the indoor plant-and-human interactions in context. These include but not limited to the effects of self-reported feelings and/or perceptions, such as naturalness, attractiveness, preference, restoration, satisfaction, and wellbeing, and the effects of objectively measured outcomes, such as task performance, physiological indicators, and health results with respect to the plant characteristics, the human characteristics, and the environmental characteristics.

Further research is needed to explore: (1) the effects of indoor plants on people's emotional states, physiological responses, attentional capabilities, cognitive functioning, behavioral activity, and health outcomes; (2) the impact of plant characteristics, such as leaf color and leaf density, on the interaction between indoor plants and humans; (3) the influence of factors such as culture, gender, age, occupation, ethnicity, and health status on these interactions; (4) the dose-response or exposure-outcome relationships between indoor plants and humans over single events, short-term periods, multiple events, or long-term periods; and (5) the mechanisms and pathways through which indoor plants affect humans.
